# Combining transcriptome analysis and GWAS for identification and validation of marker genes in the *Physalis peruviana*-*Fusarium oxysporum* pathosystem

**DOI:** 10.7717/peerj.11135

**Published:** 2021-03-22

**Authors:** Gina A. Garzón-Martínez, Francy L. García-Arias, Felix E. Enciso-Rodríguez, Mauricio Soto-Suárez, Carolina González, Aureliano Bombarely, Luz Stella Barrero, Jaime A. Osorio Guarín

**Affiliations:** 1 Centro de Investigación Tibaitatá, Corporación Colombiana de Investigación Agropecuaria Agrosavia, Mosquera, Cundinamarca, Colombia; 2 Department of Bioscience, University of Milan, Milan, Lombardy, Italy

**Keywords:** Fusarium, Cape gooseberry, RNAseq, GWAS, qPCR

## Abstract

Vascular wilt, caused by the pathogen *Fusarium oxysporum* f. sp. *physali* (*Foph*), is a major disease of cape gooseberry (*Physalis peruviana* L.) in Andean countries. Despite the economic losses caused by this disease, there are few studies related to molecular mechanisms in the *P. peruviana*—*Foph* pathosystem as a useful tool for crop improvement. This study evaluates eight candidate genes associated with this pathosystem, using real-time quantitative PCR (RT-qPCR). The genes were identified and selected from 1,653 differentially expressed genes (DEGs) derived from RNA-Seq analysis and from a previous genome-wide association study (GWAS) of this plant-pathogen interaction. Based on the RT-qPCR analysis, the tubuline (*TUB*) reference gene was selected for its highly stable expression in cape gooseberry. The RT-qPCR validation of the candidate genes revealed the biological variation in their expression according to their known biological function. Three genes related to the first line of resistance/defense responses were highly expressed earlier during infection in a susceptible genotype, while three others were overexpressed later, mostly in the tolerant genotype. These genes are mainly involved in signaling pathways after pathogen recognition, mediated by hormones such as ethylene and salicylic acid. This study provided the first insight to uncover the molecular mechanism from the *P. peruviana*—*Foph* pathosystem. The genes validated here have important implications in the disease progress and allow a better understanding of the defense response in cape gooseberry at the molecular level. Derived molecular markers from these genes could facilitate the identification of tolerant/susceptible genotypes for use in breeding schemes.

## Introduction

Plant diseases are the cause of significant crop losses in agriculture worldwide, and when combined with weeds, pathogens and pests, account for 20–30% reduced yield (Savary et al., 2019). Therefore, developing genetically improved varieties with increased yields and resistance to biotic stresses are promising alternatives for disease control (Vyska, Cunniffe & Gilligan, 2016). Members of the genus *Fusarium* represent the most widespread pathogens that cause various diseases on economically major crops (Husaini, Sakina & Cambay, 2018). The vascular wilt disease caused by *Fusarium oxysporum* has been studied in detail in some species of the Solanaceae family. For instance, in tomato, a specific *Fusarium* strain (*F. oxysporum* f. sp. *lycopersici*) is able to trigger a monogenic-type of resistance (Takken & Rep, 2010; Prihatna, Barbetti & Barker, 2018). However, considering that *Fusarium* pathogenicity is host specific, understanding the mechanisms of interaction in other pathosystems are crucial to provide alternative strategies for disease control.

Cape gooseberry (*Physalis peruviana* L.) is a valuable fruit-bearing species, recognized as a source of pharmacological compounds with anti-inflammatory, antibacterial and antitumor activities (Fang, Liu & Li, 2012; Abou Baker & Rady, 2020), as well as a functional food with vitamins A, C and B complex, minerals and antioxidant components (Puente, Nocetti & Espinosa, 2019; Ramadan, 2020). In Colombia, cape gooseberry is the second most important export fruit, mainly to European markets (Fischer et al., 2005). However, its production has decreased from 17.13 ton/ha in 2007 to 15.01 ton/ha in 2017 (MinAgricultura, 2017), mostly due to the lack of breeding materials with desirable traits such as disease resistance. Although, in Colombia, two cape gooseberry varieties (Andina and Dorada) with increased yield and reduced fruit-cracking incidence had been released, both varieties are susceptible to different pathogens (Núñez et al., 2016a, 2016b). *F. oxysporum* f. sp *physali* (*Foph*) has been identified as one of the major constraints in cape gooseberry (Simbaqueba et al., 2018). Nevertheless, the genetic basis underlying *Foph* recognition and cape gooseberry defense responses remain unclear.

Plants have different strategies to distinguish pathogens, involving different recognition layers. The first level relies on the recognition of conserved microbial elicitors known as pathogen-associated molecular patterns (PAMPs). This recognition triggers a resistance response in the plant known as PAMPs-triggered immunity (PTI) (Jones & Dangl, 2006; Choi & Klessig, 2016). When pathogens evade PTI, translocating effector proteins into the host, a second level of recognition called effector-triggered immunity (ETI) is activated. This defense level involves the detection of these effector proteins through intracellular receptors, called R proteins, which have a canonical plant nucleotide-binding, leucine-rich repeat (NLR) domain (Jones & Dangl, 2006; Van der Burgh & Joosten, 2019). Pathogen recognition through PTI or ETI triggers a whole-genome transcriptional reprograming including the synthesis of pathogenesis-related (PR) proteins. Some of the PR proteins later act by targeting pathogen cell walls or membranes, strengthening plant cell walls, and initiating hypersensitive responses that block the pathogen attack (Saboki, Usha & Singh, 2011).

Approaches such as RNA sequencing (RNA-Seq) and genome wide association studies (GWAS) have allowed the profiling of global gene expression patterns and the mapping of simple or complex traits, respectively, in both model and non-model plants. These approaches have been useful resources for the understanding of how plants respond to biotic or abiotic stimuli through the discovery of candidate genes involved in different biological processes (Iquebal et al., 2019; Sicilia et al., 2019; Safavi-Rizi, Herde & Stöhr, 2020), including those involved in plant-microbe interactions (Bartoli & Roux, 2017).

In cape gooseberry, the assembly and annotation of its leaf transcriptome was the first attempt to provide valuable resources for developing molecular tools in this species (Garzón-Martínez et al., 2012). This leaf transcriptome was used to search for proteins that encode conserved domains related to plant immunity (Enciso-Rodríguez et al., 2013). Enciso-Rodríguez et al. (2013) identified 74 immunity-related candidate genes in *P. peruviana*. These genes comprised 17 receptor-like kinase (RLKs) and 57 NLRs related to ETI, including eight and nine NLRs associated with toll/interleukin-1 receptor (TIR) and coiled-coil (CC) domains, respectively. However, this study was limited to constitutively expressed genes in leaves, which may not represent the actual mechanisms underlying the *Foph* response since it is a soil-borne pathogen. Later on, Osorio-Guarín et al. (2016) identified 17 additional candidate genes related to defense/resistance responses against *Foph* through a GWAS using 100 accessions of *P. peruviana*. More recently, Vera Alvarez et al. (2017) generated a workflow for transcriptome annotation from stem and root RNA-Seq data of the *P. peruviana*—*Foph* interaction. Nevertheless, no further analyses on candidate genes have been conducted to date. Therefore, further investigation is required to understand the *P. peruviana*—*Foph* pathosystem.

Understanding the expression patterns of critical regulatory genes contribute to elucidating the mechanisms involved in disease development and defense responses. The use of contrasting genotypes, according to their response against biotic stresses, and molecular techniques such as real-time quantitative PCR (RT-qPCR), are suitable approaches to validate the relationship between candidate genes and resistance/defense responses. RT-qPCR is a robust and cost-effective technique for quantifying messenger RNA (mRNA) (Schmittgen & Livak, 2008; Abdallah & Bauer, 2016). To date, this is the most frequently used tool to validate the molecular regulation of genes related to defense on several plant species (Użarowska et al., 2009; Casassola et al., 2015; Cregeen et al., 2015; Pombo et al., 2019).

This study aims to validate key genes identified by an RNA-Seq analysis and a previous GWAS study (Osorio-Guarín et al., 2016) of the *P. peruviana*—*Foph* pathosystem, by means of RT-qPCR. This will contribute to widening the knowledge for this orphan crop, and to help understand the resistance/defense responses in cape gooseberry. The results presented in this study will aid in the development of tolerant/resistant cape gooseberry varieties.

## Materials and Methods

### Plant material and growth conditions

One *P. peruviana* tolerant genotype (09U279-1) was used for the RNA-seq analysis based on previous studies of the differential resistance responses against *F. oxysporum* f.sp. *physali* pathogenic strain MAP5 (Enciso-Rodríguez et al., 2013; Liberato et al., 2014). Plantlets were in vitro propagated using MS medium (Murashige & Skoog, 1962), supplemented with 0.1 mg/L gibberellic acid (GA_3_) and 0.1 mg/L indole-3-butyric acid (IBA). In vitro plantlets were acclimatized in peat moss-based substrate under greenhouse conditions at 25 ± 2 °C with a 12/12h photoperiod. Three month-old plants with 10 cm height and three true leaves were transplanted to plastic bags filled with 500 g of sterilized substrate (3:1 ratio of soil:rice:husk) for subsequent inoculation experiments.

For RT-qPCR experiments, cape gooseberry genotypes, 09U128-5 and 09U140-5, previously reported as susceptible and tolerant to *Foph* (Osorio-Guarín et al., 2016), respectively, were used to validate the selected candidate genes. Plant material was in vitro propagated and grown according to the conditions mentioned above.

### Inoculation assays

The highly virulent monosporic *Foph* strain (Map5) was used for inoculum production (Enciso-Rodríguez et al., 2013). The strain was reactivated in liquid potato dextrose agar (PDA) for 8 days at 27 °C in constant agitation (120 rpm). Inoculum concentration was adjusted to 1 × 10^6^ CFU/ml according to Namiki et al. (1994). Plants were inoculated by directly applying the inoculum into the soil, while mock-inoculated plants were watered with sterile water.

### Transcriptome sequencing and in silico analysis of RNA-Seq data

Root and stem tissue from two biological replicates of an inoculated and mock-inoculated tolerant genotype (09U279-1) were collected at 0, 24, 48, 72, and 96 days post-inoculation (dpi), and flash frozen in liquid nitrogen. Samples from each time point were combined into one pool for RNA extraction, cDNA synthesis (Bio S&T Inc. Montreal, QC, Canada) and Illumina sequencing (Emory Genomics Center. Atlanta, GA, USA). RNA-Seq data from all libraries were downloaded from the Sequence Read Archive (SRA) of the National Center for Biotechnology Information (NCBI) database under BioProject ID 67621 (Table S1) (Vera Alvarez et al., 2017).

Reads were assessed for quality using FastQC version 0.11.1 (Andrews et al., 2012). Adapters trimming and quality filtering were done using Fastq-mcf from Ea-utils (Aronesty, 2011). Low quality reads with short sequence length (<50 bp) and low base sequence quality (<30) were removed. NCBI’s UniVec database (build 9.0) was used for filtering any possible contamination sequences. An in silico normalization of the filtered high quality reads was done in order to reduce the use of computational resources. Reads were normalized with a maximum coverage of reads set to 30.

Normalized reads were used for de novo assembly of the cape gooseberry transcriptome using Trinity version 2.0.2 (Grabherr et al., 2011) with default parameters. After assembly, the length distribution of assembled transcripts was compared with the well-known tomato transcriptome assembly (ITAG2.3). Furthermore, *Fusarium*-related sequences were filtered using a BLAST-Like Alignment Tool (BLAT) against a database containing the super contigs from different *F. oxysporum* strains retrieved from the *Fusarium* Comparative Database of the Broad Institute (Ma et al., 2010). *Fusarium* transcripts greater than 500 bp were removed from de novo cape gooseberry transcriptome assembly.

Two biological replicates from an inoculated tolerant genotype (SRA: SRX972116, SRX971469) and mock-inoculated read libraries (SRA: SRX980678, SRX978916) were mapped to the de novo cape gooseberry transcriptome assembly for transcript abundance estimation using the Trinity tool kit (Garber et al., 2011) with the RSEM method (Li & Dewey, 2011). Differentially expressed genes (DEGs) across both inoculated and mock-inoculated plants were identified using the edgeR package version 3.0 (Robinson, McCarthy & Smyth, 2009). Transcripts with a fold change >2 and a false discovery rate (FDR) < 0.01 were considered as significant DEGs. Functional annotation and gene ontology (GO) analyses were carried out by a local BLASTX search against the UniprotKB/Swiss-Prot and the tomato database (ITAG2.3), using an expected value threshold of 1e−^3^. BLAST files were filtered and parsed for the best high score match using the perl script BlastAddDescriptor (Bombarely, 2012).

### Candidate genes selection and primer design

From the DEGs identified, candidate genes were selected for further RT-qPCR validation assays based on their functional annotation. The closest tomato gene (Build SL3.0) to the DEGs was used for designing primers after identifying the exon-intron boundaries from the tomato genes using the IDT tool (Integrated DNA Technologies, 2018). Genes were named using the corresponding tomato ID used for the primer pairs design.

Furthermore, seven candidate genes, flanking genomic regions associated with resistance/defense to *Foph*, were selected from a previous GWAS study of the *P. peruviana*—*Foph* interaction (Osorio-Guarín et al., 2016). The primer design strategy consisted of the alignment of genotyping-by-sequencing (GBS) reads from the candidate genes, of about 64 bp, against the de novo cape gooseberry transcriptome. Primers were designed from selected *Physalis* transcript sequences using Primer3 (Rozen & Skaletsky, 2000). Amplicon sizes were confirmed using 2% (w/v) agarose gel stained with SYBR Safe (1X) (Invitrogen, Waltham, MA, USA). Genes were named according to the corresponding *Physalis* transcript ID used for the primer pairs design.

The pyrophosphatase (*PPA2*), β-tubulin (*TUB*), actin (*ACT*) and elongation factor (*EF-1α*) were evaluated as reference genes as previously reported in different RT-qPCR analyses (Czechowski et al., 2005; Hong et al., 2010). The selection of the best reference gene for cape gooseberry was based on analyses of melting curves with four-fold serial dilutions (1:1, 1:4, 1:16 and 1:64) and by calculating their variation among samples.

### RT-qPCR assay

Forty plants from the susceptible (09U128-5) and tolerant (09U140-5) genotypes selected were inoculated with the *Foph* strain MAP5. Ten plants were mock-inoculated using sterile water. Plants were arranged in a completely randomized block design with two replicates (10 plants per replicate) per treatment and genotype. Inoculation assays were done at the Tibaitatá Research Center of the Corporación Colombiana de Investigacion Agropecuaria (Agrosavia).

Plant symptoms were scored according to the disease severity scale described by Enciso-Rodríguez et al. (2013) with some modifications (Table S2). The severity scale of the disease consisted of five degrees. These levels were: 0: plant without symptoms, 1–4: plant with increasing degrees of wilting and 5: dead plant. Root tissue was collected at 1, 3 and 5 degrees of the scale. To avoid conflating the disease with nutrient deficiency symptoms, plants were fertilized with 2 ml/L of foliar fertilizer and a 10-20-10 edaphic fertilizer. All samples were immediately frozen in liquid nitrogen and stored at −80 °C for RNA extraction.

### RNA isolation and cDNA synthesis

Total RNA was isolated from root tissue using the Plant RNA Purification Kit (Qiagen, Hilden, Germany), according to the manufacturer’s instructions. RNA samples (1 μg) were treated with DNase TURBO (Invitrogen, Waltham, MA, USA) to discard contaminating DNA. The purified RNA was quantified using a NanoDrop 2000 UV-Vis spectrophotometer and visualized in 1.5% (w/v) agarose gel. Complementary DNA (cDNA) synthesis was performed using 500 ng of total RNA with the iScript™ cDNA Synthesis Kit (Bio-Rad, Hercules, CA, USA) according to the manufacturer’s instructions.

### RT-qPCR analysis

Real-time quantitative PCR reactions were performed on an iCycler IQ5 real-time PCR System using the iQ SYBR Green supermix System (Bio-Rad, Hercules, CA, USA), with the following thermocycler conditions: one cycle of initial denaturation at 95 °C for 3 min, followed by 40 cycles of 95 °C for 15 s and a final extension at 56 °C for 60 s in a 10 µl final volume. Each reaction was performed in triplicate. Melting curves were generated at 65–95 °C after 40 cycles to check for primer pairs specificity.

Finally, the 2^−ΔCT^ method was used to compare the differential expression from each candidate gene at the three degrees of the disease scale from the tissue collected. One-way ANOVA followed by Tukey HDS for multiple pairwise comparisons were applied. The gene expression profiling was done using the following equation (Schmittgen & Livak, 2008):

2^−ΔCT^ where ΔC_T_ = (C_T_ gene of interest – C_T_ reference gene)

## Results

### *P. peruviana* transcriptome assembly and DEGs profiling

A reference transcriptome was assembled for cape gooseberry, as is common for non-model organisms. Here, we used 98 million reads (Table S3), with an average length of 100 bp, to generate a de novo transcriptome assembly from previous data (Vera Alvarez et al., 2017) and subsequent identification of DEGs from the *P. peruviana*—*Foph* pathosystem. After trimming adapters, primers, contaminants and low-quality sequences, a total of 87,488,110 reads were used for an in silico normalization process, which resulted in 17,664,763 sequences used for a de novo transcriptome assembly (Table S3). Based on the assembly’s information provided by the assembly, a total of 60,934 transcripts with a read length greater than 500 bp and an N50 of 1,511 bp, were generated (Table S4). After extracting *F. oxysporum* sequences, 59,476 transcripts were used as reference for mapping reads for downstream differential expression and functional analysis.

**Figure 1:**
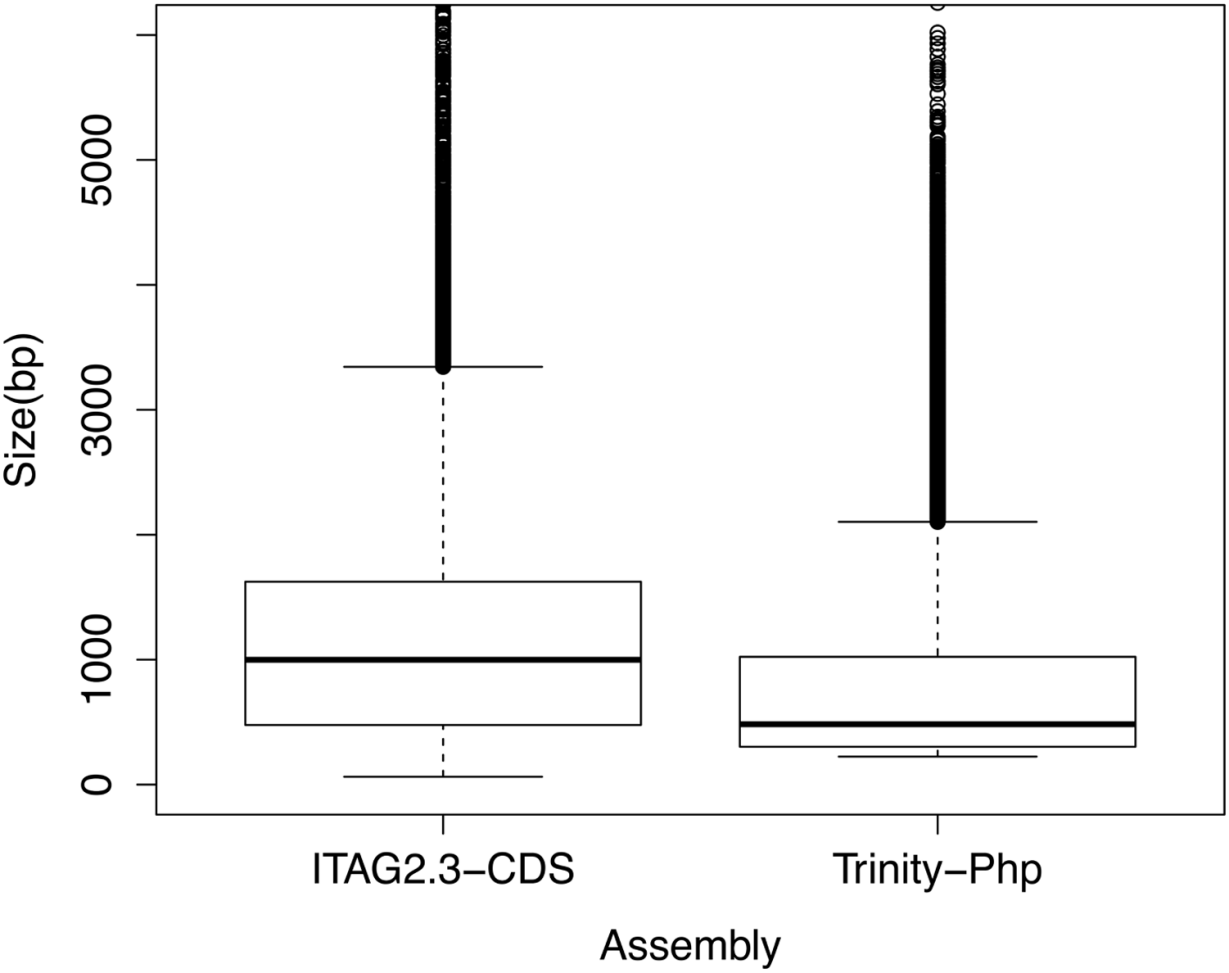
Boxplot comparison of the de novo assembled transcripts length distribution using the Trinity software. First box indicates tomato cDNA gene models (ITAG2.3 CDS) and second box represents Trinity de novo assembly from *P. peruviana* (Trinity-Php). Statistical significance between both transcripts length means was analyzed by a *t*-test, *p* < 0.01.

To assess the quality of the transcriptome assembly, the length distribution of assembled transcripts was compared to gene models of a well-annotated closely related species (*Solanum lycopersicum*), as done by Villarino et al. (2014) (Fig. 1). Even though, it was observed a significant difference (*p < 0.01*) between the mean size distribution of all *P. peruviana* trinity transcripts (125,590 sequences, mean size transcripts 779 bp) and the tomato full annotated transcriptome (34,727 sequences, mean size transcripts 1,208 bp) (Fig. 1), the N50 value of *P. peruviana* assembly (N50 25,226 sequences with 1,207 bp average length) was close to the tomato N50 annotated transcriptome (N50 7000 sequences with 1,400 bp average length).

**Figure 2:**
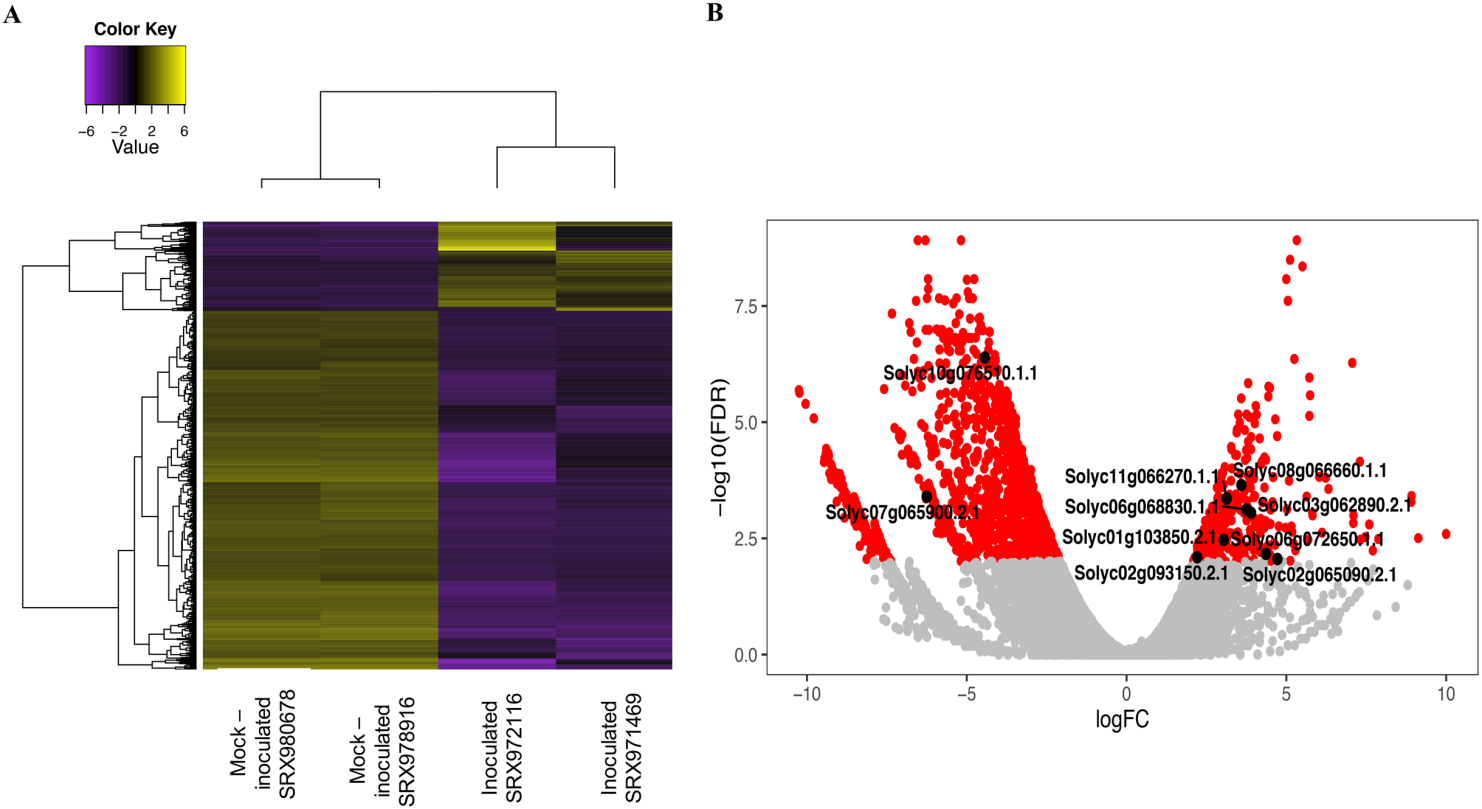
Heat map and volcano plot of differential expressed transcripts from two biological replicates of a tolerant genotype inoculated and mock-inoculated read libraries. (A) The yellow color represents upregulated transcripts and the purple color indicates down-regulated transcripts from both inoculated and mock-inoculated libraries. (B) The red dots indicates differentially expressed genes, while the gray dots represents genes that are not differentially expressed between the inoculated and mock-inoculated libraries. Black dots indicate the 10 candidate genes selected for further study.

Next, four transcriptome libraries were analyzed to find DEGs related to the *P. peruviana*—*Foph* pathosystem. When comparing inoculated and mock-inoculated libraries, 329 up-regulated transcripts and 1,323 down-regulated transcripts were found (Fig. 2A; Data S1). A gene ontology analysis was done to determine the functions of the DEGs (Fig. S1; Table S5). GO classification showed that the 1,652 DEGs were classified in at least one GO term at level 2 (molecular function, biological process or cellular component). In the molecular function category, binding (GO:0005488) and catalytic activity (GO:0003824), were the most abundant GO terms. The biological process category revealed different ontologies, including cellular process (GO:0009987), response to stimulus (GO:0050896) and metabolic process (GO:0008152). Finally, for cellular component, transcripts involved in cell (GO:0005623) and cell part (GO:0044464) were the most highly represented (Fig. S1).

### Candidate gene selection and primer design

Based on the DEGs, 10 candidate genes associated with plant defense responses were selected (Fig. 2B; Table 1). These genes included transcription factors like Apetala2/Ethylene Response Factors (AP2/ERFs), patatin, Xyloglucan Endotransglucosylase/Hydrolase (XTHs), among other genes which are known to be involved in responsive pathways related to stress regulation (Olsen, Popper & Krause, 2016; Najafi, Sorkheh & Nasernakhaei, 2018; Cheng, Song & Wu, 2019). On the other hand, seven candidate genes from a previous GWAS study (Osorio-Guarín et al., 2016) were selected based on (1) mapping to the tomato genome genes with a complete functional annotation and (2) a functional annotation related to defense responses (Table 1). Among these genes, the WD40 repeats, legume lectines, G proteins, and others were included (Smith et al., 1999; Roopashree et al., 2006; Trusov et al., 2009).

**Table 1:** Selected candidate genes identified in RNA-Seq and a preliminary GWAS study and primers sequences for RT-qPCR analysis. ID correspond to the tomato gene ID and *Physalis* transcript ID used for the primer pairs design.

ID	Functional annotation	Primer sequences	Tm (°C)	Size (bp)	Source
*Solyc01g103850.2.1*	Ubiquitin-like domain-containing CTD phosphatase	F-TCGACTGCAAGCCTTTAGG	55.5	148	DEGs
		R-GCGTGTGCCTTTCTGAATG			
*Solyc02g065090.2.1*	Patatin-like protein 3	F-TTGATGGAGGTGTAGCTGC	55.3	142	
		R-CCTGTGCCTAGTGATAGAACC			
*Solyc02g093150.2.1*	AP2-like ethylene-responsive transcription factor	F-ATTCACTGGCATGTATCCTGG	54.8	149	
		R-AGTACTGGACACGAACTTGC			
*Solyc03g062890.2.1*	Superoxide dismutase [Cu-Zn] 2	F-GGAACGATAGTGTGCAAGGATC	55.6	136	
		R-CCGTTAGTGGTATCACCCAAG			
*Solyc06g068830.1.1*	Ethylene-responsive transcription factor ERF115	F-GCAAAACCAATTACCTCAGCTC	54.4	150	
		R-AAAGTACCCAACCATACACGAG			
*Solyc06g072650.1.1*	Auxin responsive SAUR protein	F-GCCAAATATGCAAACCATCC	53.0	217	
		R-CTCCATGTCCCAAGACCCTA			
*Solyc07g065900.2.1*	Fructose-bisphosphate aldolase	F-CTTAGTGGAATTATCTTGTTCGAGG	52.7	141	
		R-GGGTAGTTGTCTCACCATTGG			
Solyc08g066660.1.1*	Ethylene-responsive transcription factor ERF096	F-CACGTATTTGGCTTGGGACT	55.3	157	
		R-TGAATGTCACGCGGACTAAG			
*Solyc10g076510.1.1*	Pyruvate decarboxylase 1	F-CACAACGGGCAAGGAAAATG	55.6	139	
		R-TGCTGGTGTCATCCTTGTG			
*Solyc11g066270.1.1*	Xyloglucan endotransglucosylase/hydrolase 9	F-CCTTCTAGCTCTCCTTCGGG	55.5	76	
		R-AGTTCCTATGCACCCACAAC			
		R-GGGTAGTTGTCTCACCATTGG			
Php_TR3359*	Proteasome subunit beta type, proteinaceous elicitor of plant defense reactions	F-GATCCCACAGCATCATAAGTGA	55.8	119	Osorio-Guarín et al. (2016)
		R-CAGCTGCTTTCGAACACACTAT			
Php_TR3653*	Legume lectin beta domain, signaling molecule in defense response	F-ATGCAAATGTTGGAACAATCAG	51.4	92	
		R-TTCTCCTTTTTCGGTTCTTCTG			
Php_TR7902*	WD-40 repeats, signal transduction and hypersensitive response	F-TTGCTGACTTCCATGAGCTTTA	52.6	103	
		R-CCAGGCAATAGATGTTTGATGA			
Php_TR8981*	Lipase, class 3, related to PAD4, EDS1 y SAG101 (Accumulation of salicylic acid)	F-TTTCATCCTCACCACACTATGC	55.0	103	
		R-GAGGACAGAAATCCACCATCTC			
Php_TR46393*	G-protein-coupled receptor, immunity in plants	F-AAACAGGACACACAGGAAAGGT	53.2	115	
		R-GCATTGTTTAGCATCCGTATCA			
Php_TR64396*	Thioredoxin domain, oxidative stress tolerance	F-TTACCCTTACTGGCGATACGTT	51.4	101	
		R-CAGGTTCTTGCAGTGCTTACAC			
Php_TR69681*	Major facilitator superfamily transporter, exporting toxins	F-GCTGTTGTTGTTCAGATGGAAA	53.6	89	
		R-CCTCTATCGCACATATCCACAA			
*Elongation factor (EF)*	–	F-TGGTTTTGAAGCTGGTATCTCC	54.6	140	Reference Genes
		R-CATACCTAGCCTTGGAGTACTTG			
*Pyrophosphatase (PPA2)*	–	F-GATGAGTTTCCTGATGTTCGTTTG	54.0	136	
		R-GAACCCTCCAGTGTCTATCTTC			
*Tubulin (TUB)*	–	F-GTCTGGTGCTGGAAACAATTG	54.1	133	
		R-TGAATGGCACACTTGAAAACC			
*Actin (ACT)*	–	F-GTACAGTGTCTGGATTGGAGG	54.7	73	
		R-GCCCTTTGAAATCCACATCTG			

**Notes:**

* Set of genes evaluated by RT-qPCR.

Tm, melting temperature; bp, base pair.

**Figure 3:**
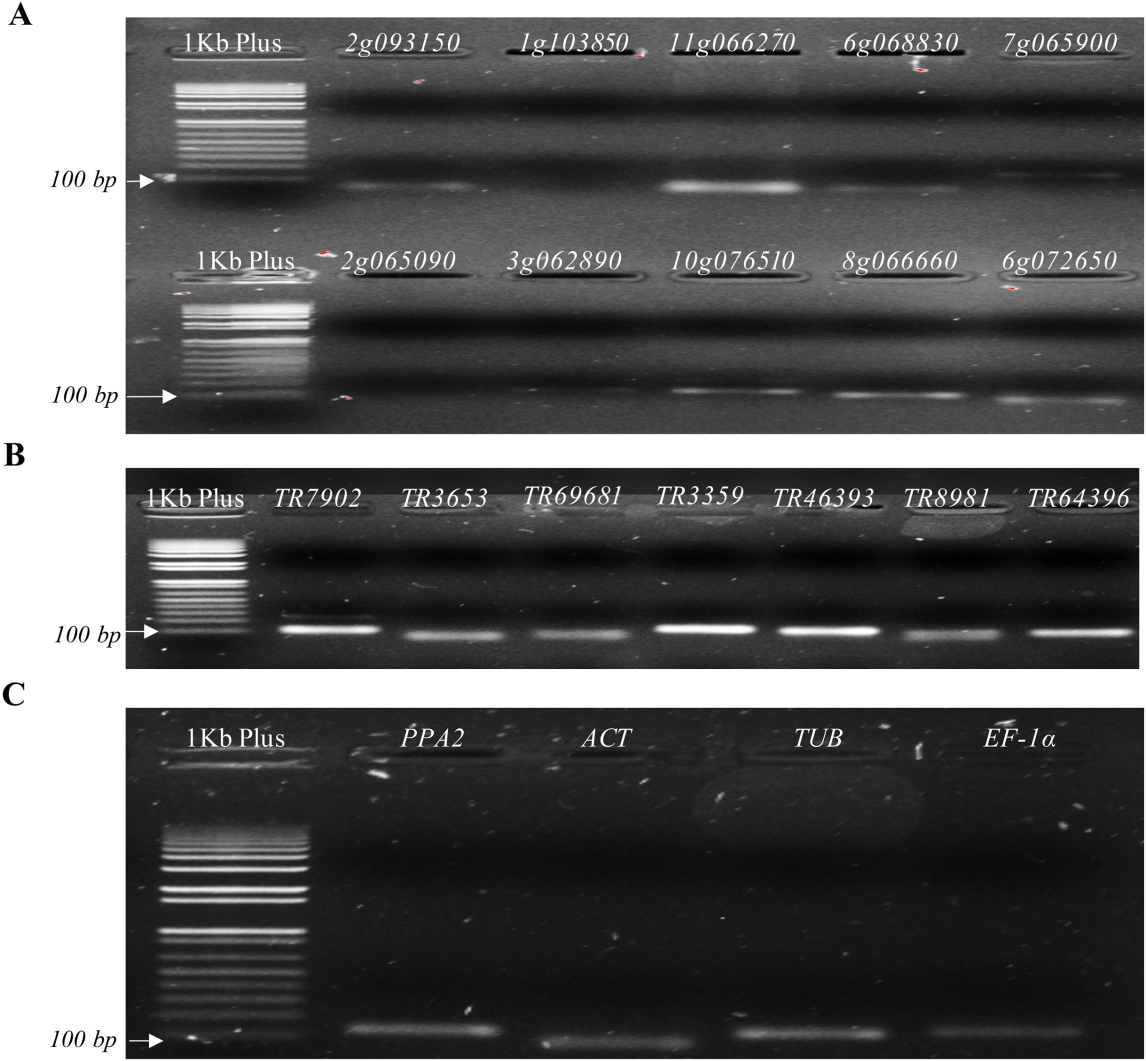
Agarose gels from candidate and reference genes selected. (A) Genes selected from the differentially expressed genes (DEGs) analysis. (B) Genes selected from Osorio-Guarín et al. (2016). (C) Reference genes.

A BLAST comparing the 10 DEGs primer pairs sequences to the de novo *P. peruviana* transcriptome showed a partial alignment (∼10 bp from 20 pb), mismatches between primer pairs and transcripts (∼3 bp), as well as secondary annealing of the primer pairs with different cape gooseberry transcripts (Table S6). In addition, electrophoresis in 2% (w/v) agarose gels from the 10 DEGs primer pairs showed that some primer pairs amplified a single PCR product with the expected target size (*Solyc11g066270.1.1, Solyc10g076510.1.1, Solyc08g066660.1.1, Solyc06g072650.1.1*), while others did not amplify (*Solyc01g103850.2.1, Solyc02g065090.2.1, Solyc03g062890.2.1*) or showed a weak amplification (*Solyc02g093150.2.1, Solyc06g068830.1.1, Solyc07g065900.2.1*) (Fig. 3A). When evaluating a melting curve of three out of 10 of the genes with the best BLAST results, we found a low specificity of two primer pairs (*Solyc06g068830.1.1, Solyc11g066270.1.1*) and a strong specificity for one (*Solyc08g066660.1.1*) (Figs. S2A, S2B and S2C). Considering these results, we decided to continue with the candidate gene *Solyc08g066660.1.1*, which showed good results in the BLAST alignment and melting curve.

In contrast, agarose gels from the seven primer pairs selected from a GWAS study (Osorio-Guarín et al., 2016) showed high specificity and the expected target size (Fig. 3B), which was corroborated with their melting curves (Figs. S2D, S2E and S2F). No amplification was observed in control samples without reverse transcriptase, cDNA template or primers in the RT-qPCR reaction. Based on the results of both primer design strategies, eight candidate genes, one from DEGs and seven from the previous GWAS study (Osorio-Guarín et al., 2016) were used for further RT-qPCR analysis.

Electrophoresis in 2% (w/v) agarose gels revealed that all the reference genes (*PPA2*, *TUB, ACT* and *EF-1α*) amplified a single product with the expected size (Table 1; Fig. 3C). Expression patterns of these four genes in a sample set of inoculated plants were quantified using RT-qPCR. Standard curves revealed a high primer specificity for all selected genes, presenting a good fluorescence quality and primer dimer absence (Fig. S3C). The genes *TUB* and *PPA2* with a similar melting curve temperature of 80 °C were selected to determine which of the two primer pairs showed the least variation between different cDNA samples from the assay. It was found that the reference gene *TUB* had greater stability and less variation in C_T_ values with a range of −0.767 to −0.131 and −0.004 to −0.893 for both biological samples, while *PPA2* showed a greater variation ranging from −1.9 to −10.8 and −0.360 to −1.312. Therefore, the *TUB* reference gene was selected for subsequent analyses.

### Phenotypic differences between selected genotypes in response to *Foph*

Cape gooseberry genotypes 09U128-5 and 09U140-5 were inoculated with the highly virulent *Foph* strain Map5. The first phenotypic symptoms were observed at 17 days post-inoculation (dpi) for the susceptible genotype 09U128-5, presenting small yellow spots (chlorosis) on the leaves. At 25 and 30 dpi, this genotype exhibited symptoms associated to scale degrees 3 and 4, respectively, according to the disease severity scale, to finally collapse at 35 dpi. On the other hand, the tolerant genotype 09U140-5 started to present the first symptoms on leaves at 24 dpi, later than the susceptible genotype. At 30 and 40 dpi, this genotype exhibited symptoms related to scale degrees 3 and 4, respectively. This genotype died at the end of the experiment (60 dpi). The tolerant genotype took 25 days longer to die compared to the susceptible genotype (Fig. S4). No symptoms were observed in the mock-inoculated plants.

### Validation of candidate genes using RT-qPCR

The eight candidate genes selected were evaluated using the aforementioned cape gooseberry genotypes exposed to *Foph*. Plant material was collected at three different scale degrees of the disease to ensure a broad spectrum of the scale in the expression profile. The *TUB* gene was used as the reference gene to normalize the data and determine the relative expression level of the candidate genes.

**Figure 4:**
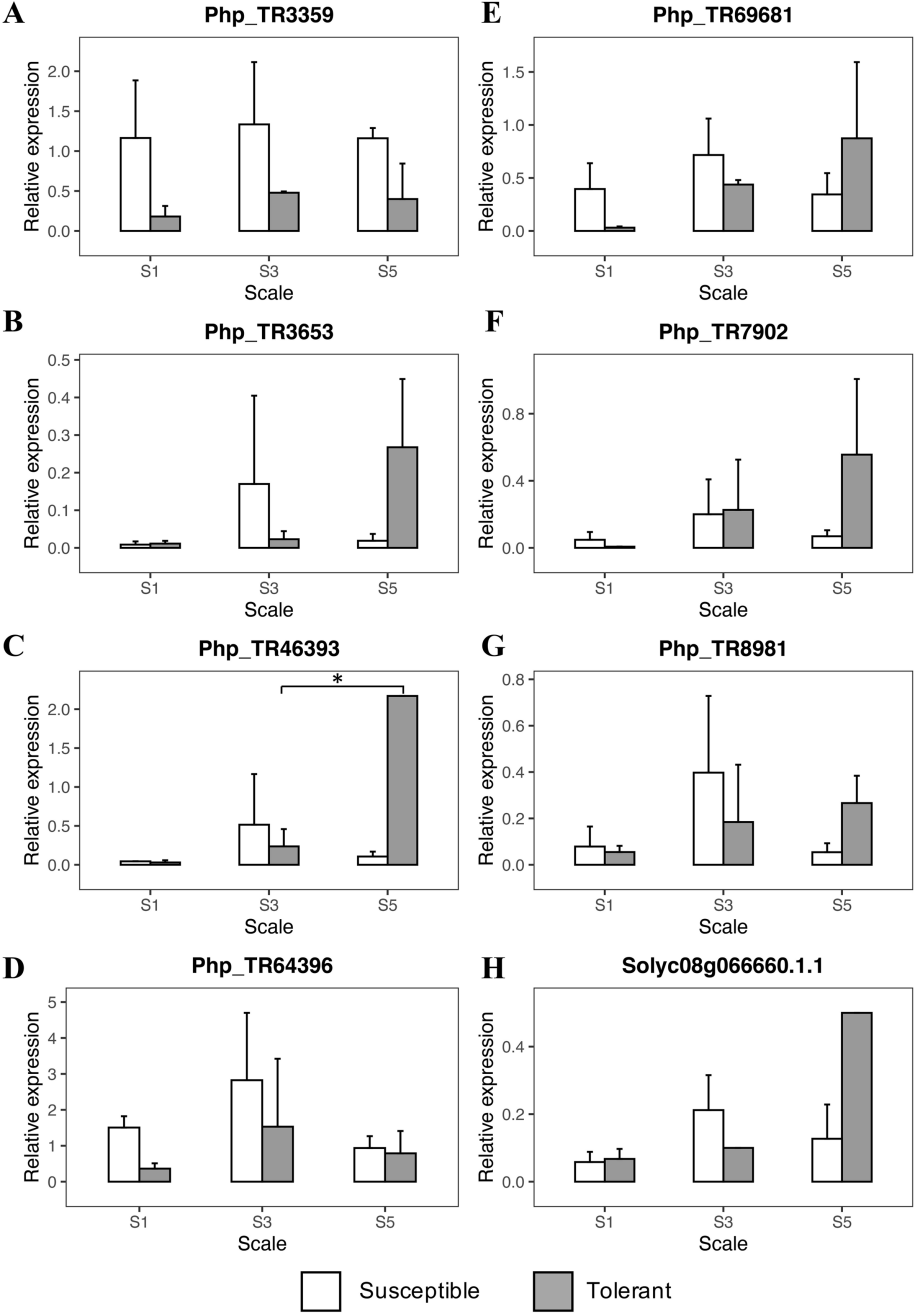
Quantitative real-time PCR (qRT-PCR) analyses of eight candidate genes related to *Foph* resistance response in cape gooseberry root tissue. Genes: (A) PhpTR3359, (B) PhpTR3653, (C) PhpTR46393, (D) PhpTR64396, (E) PhpTR69681, (F) PhpTR7902, (G) PhpTR8981, (H) Solyc08g066660.1.1 Asterisk indicates the significant diference of the tolerant genotype among the disease severity scale (One-way ANOVA, Tukey HSD test, *p* < 0.05).

The RT-qPCR analysis showed an initial trend in the scale degrees 1 and 3 from the genes *Php_TR3359*, *Php_TR64396*, and *Php_TR69681* with an increased response to the initial infection stages of *Foph* in the susceptible genotype (Fig. 4; Table S7). Once the disease progressed to scale degree 5, the transcript level of genes, mainly related to signal transduction and hormone-regulated induced defense responses (ethylene and salicylic acid), increased in the tolerant genotype (*Php_TR8981, Php_TR46393*, *Solyc08g066660.1.1*) (Fig. 4). On the other hand, the expression profile of *Php_TR3653* and *Php_TR7902* increased at the scale degree 5, in the tolerant genotype. Overall, as the disease progresses, the plant triggered a stronger response against *Foph*, which extends the lifespan of the tolerant plant.

## Discussion

### Candidate defense-related genes in the *P. peruviana—Foph* pathosystem were selected through DEGs and GWAS strategies

Understanding the molecular mechanisms by which plants respond to their environment has been key to tackling biotic and abiotic stresses in plants. The development of next-generation sequencing (NGS) technologies has accelerated the identification of genes associated with agronomic-related traits for important crops (Nguyen et al., 2019), enabling the earlier selection of progenies with desirable attributes through marker-assisted selection (MAS). In this study, we used the results from two different NGS approaches, RNA-Seq and GWAS, to identify and select candidate genes associated with the *P. peruviana*—*Foph* pathosystem.

RNA-Sequencing has been widely used for analyzing gene expression patterns in different tissues and developmental stages under various conditions (Tsukagoshi et al., 2015; Brito et al., 2017; Howlader et al., 2020). Here, we analyzed the data of previously reported transcriptome libraries (Vera Alvarez et al., 2017) from cape gooseberry’s root and stem tissues derived from the interaction with *Foph*. A comparison between a de novo cape gooseberry transcriptome assembly with the tomato transcriptome, as the most closely related species from the Solanaceae family with full annotated transcriptome, showed a similar distribution. According to Villarino et al. (2014), this means an accurate assembly approach. After the assembly, the DEGs were screened using a comparative analysis of two libraries, corresponding to the *Foph* inoculated tolerant genotype (SRA: SRX972116, SRX971469) and its mock-inoculated (SRA: SRX980678, SRX978916) plants. Previous studies have used the cape gooseberry’s leaf transcriptome (Garzón-Martínez et al., 2012) to identify immunity-related genes and derived markers (Enciso-Rodríguez et al., 2013). This is the first root/stem specific study that aims to identify candidate genes related to the response of cape gooseberry to *Foph*.

In this study, 10 DEGs and seven candidate genes, reported from a previous GWAS study, have the characteristic gene architectures from genes involved in downstream recognition pathways related to biotic stresses. Some DEGs were associated with gene ontologies such as response to stress with the typical conserved domains related to plant immunity such as kinase domains. These domains are well known for being involved in downstream signaling (i.e., mitogen-activated protein kinases) after pathogen recognition through either PTI or ETI (Jones & Dangl, 2006) We also found candidate genes with associated domains such as AP2, ubiquitin and patatine-like domains, as well as: plant auxin responsive proteins, glycosyl hydrolases proteins, superoxide dismutase, and others (Table 1). In general, these candidate genes are involved with the expression or synthesis of defense-related compounds, triggered by a possible *Foph* recognition through either PTI or ETI in *P. peruviana* as reported in other species (Dreher & Callis, 2007; Minic, 2008; Bielach, Hrtyan & Tognetti, 2017; Subki, Abidin & Yusof, 2018; Najafi, Sorkheh & Nasernakhaei, 2018; Cheng, Song & Wu, 2019).

### RT-qPCR standardization allowed for reliable verification of gene expression in the *P. peruviana—Foph* pathosystem

Real-time quantitative PCR has been a valuable tool for a cost-efficient validation of candidate gene expression. However, quantification of gene expression is affected by various experimental sources of variation that need to be carefully controlled, such as primer performance, the quantity of the initial material, sample-sample variation, the quality of the RNA, the reference genes used, the experimental design and the statistical methods employed for the analysis (Chen et al., 2011; Bustin & Huggett, 2017).

For designing primers, two approaches were used: (1) homologous sequences of the tomato transcriptome for the DEGs genes and (2) de novo cape gooseberry transcripts for genes or genomic regions derived from GWAS. As expected, the use of the de novo cape gooseberry transcriptome resulted in primer pairs with a higher specificity than the ones designed from tomato gene models. Despite the fact of the high degree of synteny in terms of gene order, orientation and exon/intron structure in the Solanaceae family (Frary, Doganlar & Frary, 2016), chromosomal rearrangements/mutations must be present between the two species, resulting in sequence variation within exon/intron boundaries used for the design of primers from DEGs. Thus, the primer pairs may not match as specifically as when using short sequences (tags) and transcripts from the same species. In addition, various genomic elements and gene features can impact primer specificity. Primers may inadvertently cross-match to non-target transcripts or other members of a gene family (Yuen, Brooks & Li, 2001; Ruiz-Villalba et al., 2017) or to similar but untargeted paralogous sequences such as intron-less pseudogenes (Dapprich et al., 2016). This is particularly uncertain in *P. peruviana* which currently lacks a reference genome sequence. Thus, plants present a large variety of mRNAs which can be very related and induced to the wrong amplification (Tian et al., 2020). In this study, from the 17 primer pairs designed, eight showed a high efficiency and were selected for further validation. A re-design from the primers for the remaining DEGs using the de novo *P. peruviana* transcriptome and further RT-qPCR analyses need to be considered.

In order to select appropriate reference genes, which are essential for the normalization of the expression of a target gene (Kozera & Rapacz, 2013), four genes were evaluated. Reference genes are those associated with basic cellular functions and are routinely used due to their expression at relatively constant levels in different organs, developmental stages and different stress conditions. However, a range of variability is expected depending on their stability and expression level under certain biological conditions (Hong et al., 2010; Kozera & Rapacz, 2013; Curis et al., 2019). This study is the first that evaluated different reference genes for *Foph* response in cape gooseberry. Even though most studies use traditional reference genes (*Actin*, *EF*), they are not always stably expressed in different species or experimental conditions (Rebouças et al., 2013; Zhu et al., 2013). Here, from the four genes evaluated, the *TUB* gene presented the lowest variation among samples. Osorio-Guarín, García-Arias & Yockteng (2019), also recommended *TUB* as an endogenous gene to evaluate the phytoene desaturase (*PDS*) gene expression in cape gooseberry leaf tissue, confirming the suitability of this gene for RT-qPCR analyses. This gene is also known to show a highly stable expression in carrot (Tian et al., 2015), sisal (Sarwar et al., 2020) and soybean (Hu et al., 2009).

### Contrasting phenotypic responses between selected genotypes in response to *Foph* allowed proper sampling for RT-qPCR analysis

For the experimental design, it was important to follow the disease progress to ensure proper sampling of genotypes’ responses to *Foph*. The use of a modified severity scale from Enciso-Rodríguez et al. (2013), helped us to clearly separate the plant’s reponse to *Foph*, which it is suppported by Manzo et al. (2016), who recommended longer evaluation times, considering that the response to *F. oxyxporum* disease in a closely related species like tomato is slow.

The greenhouse assay showed similar responses to *Foph* as in a previous study (Osorio-Guarín et al., 2016). In the tolerant genotype, the vascular wilt disease was gradually increased with time, exhibiting a delay in the disease progress, dying at the end of the experiment. This delay in the infection suggests that the tolerance response against *Foph* was mediated by minor genes involved in horizontal or polygenic-related resistance, which also implies a reduction in the severity of the symptoms when compared with the susceptible genotype (Pagán & García-Arenal, 2018).

### Verification of candidate gene expression in the *P. peruviana—Foph* pathosystem by RT-qPCR shed light on the interaction for crop improvement

During the first stages of the infection, *Foph* elicited the expression of the *Php_TR3359*, *Php_TR69681* and *Php_TR64396* candidate genes in the cape gooseberry susceptible genotype. Specifically, *Php_TR69681* (Tomato gene ID: *Solyc05g051900.2*) is related to the major facilitator superfamily transporter proteins (MFS). MFS play an important role in controlling the exchange of toxins governing plant-pathogen interactions by exporting the plant pathogen toxins outside the cell (Peng et al., 2011; Menke, Dong & Kistler, 2012). The *Zm-mfs1* defense-inducible gene that encodes a protein related to MFS transporters in maize, appears to be part of the initial defense response to fungal pathogen infection. It subsequently displays an expression decline when symptoms are developed in either susceptible or resistant genotypes (Simmons et al., 2003). In the present study, this gene showed a lightly increased expression at the earlier stages of the infection, with a subsequent decrease when the plant was approaching death in the susceptible genotype, which was opposite to the gene response in the tolerant genotype. On the other hand, the gene *Php_TR3359* (Tomato gene ID: *Solyc02g084920.2*) is related to the alpha/beta subunit of the proteasome system, which according to Suty et al. (2003), it translates signals that trigger the systemic acquired response (SAR) against pathogens’ attack. Here, an earlier induction of this gene was observed in the susceptible genotype, lasting until the plant collapse. Similarly, Üstün et al. (2016) showed that proteasome activity is strongly induced during basal defense in *Arabidopsis thaliana*, being an essential component of molecular pattern-triggered immunity and SAR induction. The third gene, *Php_TR64396* encodes thioredoxins proteins that act as antioxidants, playing an essential role in tolerance to oxidative stress and are involved in defense mechanisms to the mosaic virus in tobacco (Tomato gene ID: *Solyc11g069690.1*) (Vieira Dos Santos & Rey, 2006; Sun et al., 2010). This gene was also induced in the earlier stages of the disease with a high expression in the degree scale 3. All the previous genes were expressed in the susceptible genotype, and although they had been previously associated with defense response in plants, they could also be associated to susceptibility as observed in potato and Arabidopsis. For instance, the defense-associated gene *DMR6* is expressed during the downy mildew infection in Arabidopsis, favoring pathogen attack and had also been associated with susceptibility responses in potato (Van Damme et al., 2008; Sun et al., 2016). Since the expression of *Php_TR64396* and *Php_TR3359* are highly induced in the susceptible cape gooseberry genotype during *Foph* infection, it is possible that these genes could be associated to susceptibility-related responses (Fig. 4). However, caution must be taken when analyzing the role of these genes in the *Foph* susceptible genotype. These genes are derived from previously associated genomic regions detected by GWAS (Osorio-Guarín et al., 2016), and likely co-segregate with the actual causal gene located in the same linkage disequilibrium block.

Interestingly, a second group of candidate genes (*Php_TR46393*, *Php_TR8981*, *Solyc08g066660.1.1*), involved in hormone synthesis pathways, presented an induction in later stages of the infection, mainly in the tolerant genotype. The *Php_TR46393* gene (Tomato Gene ID: *Solyc08g61260.2*) is related to a large family of transmembrane receptor proteins called G-protein-coupled receptors (GPCRs) in fungi and metazoans. These genes are believed to be involved in plant defense signaling by mediating responses initiated by multiple RLKs (Liu et al., 2013), and are associated with resistance to necrotrophic pathogens such as *F. oxysporum*, *Alternaria brassicicola* and *Botrytis cinerea* (Trusov et al., 2006, 2009). Trusov et al. (2009) showed in later phases of the disease response that heterometric G proteins enhanced the expression of jasmonic acid (JA)/ethylene (ET)-dependent genes conferring resistance to *F. oxysporum* in Arabidopsis. These observations suggest that G proteins might play a key role in the tolerance response to *Foph* in cape gooseberry. The gene *Php_TR8981* (Tomato Gene ID: *Solyc09g098450.2*), also associated with stress response in plants, encodes a class 3 lipase protein and has a relationship with the proteins PAD4, EDS1 and SAG101 that form a systemic signal that functions as the main barrier against pathogens, involving SA accumulation (Wiermer, Feys & Parker, 2005). Finally, the *Solyc08g066660.1* gene encodes ethylene response transcription factor (ERF), which act either as an activator or repressor of plant defense responses (Thirugnanasambantham et al., 2015). Overexpression of ERFs has been shown to enhance plant resistance to *F. oxysporum* (Berrocal-Lobo & Molina, 2004). In general, the overexpression of these genes in the tolerant *P. peruviana* genotype clearly shows the late response to *Foph* infection. This response involved the pathogen recognition and posterior signaling cascade that induces local and systemic responses mediated by plant hormones such as ET and SA.

Other important genes that were expressed in the tolerant genotype, at later stages, were *Php_TR7902* and *Php_TR3653*. The first one (Tomato Gene ID*: Solyc08g081990.2*) encodes a WD-40 repeat involved in different functions, including the hypersensitive response (Smith et al., 1999). The second gene *Php_TR3653* (Tomato Gene ID: *Solyc12g049500.1*) has a beta domain of legume lectin, that according to Roopashree et al. (2006) might be involved in the protection against pathogenic agents by producing lipoxygenases. Specifically, legume-like lectin receptor kinases are involved in sensing cell wall integrity and defense response to *Phytophthora infestans* (Bouwmeester et al., 2011).

Overall, harnessing genomic regions associated with *Foph* resistance/susceptibility responses in cape gooseberry will contribute to the establishment of appropriate breeding schemes for early selection of resistant/tolerant genotypes. The validation of candidate genes identified from RNA-Seq and GWAS will contribute to this goal, accelerating the generation of new cape gooseberry varieties with desirable attributes and the understanding of *P. peruviana* responses against *Foph*.

## Conclusions

This is the first attempt to validate a set of resistance/defense response candidate genes during *P. peruviana*—*Foph* interaction using RT-qPCR. This study corroborates the differential expression of eight candidate genes at different stages of the *Foph* disease severity scale. These genes could be used for developing specific markers to identify susceptible and tolerant cape gooseberry cultivars against *Foph*. Thus, these genes are great candidates for breeding purposes and marker-assisted or genomic selection programs. Further, analysis of more additional DEG candidate genes would provide a broader understanding of this pathosystem.

## Supplemental Information

10.7717/peerj.11135/supp-1 Supplemental Information 1 Gene ontology classification of differentially expressed genes (DEGs). (A) Molecular Function. (B) Biological process. (C) Cellular component.

10.7717/peerj.11135/supp-2 Supplemental Information 2 Melting curves from three differentially expressed genes (DEGs) and three candidate genes selected from a previous GWAS study (Osorio-Guarín et al., 2016). (A) DEG: Solyc06g068830.1.1. (B) DEG: Solyc11g066270.1.1. (C) DEG: Solyc08g066660.1.1. (D) GWAS: Php_TR7902. (E) GWAS: Php_TR3653. (F) GWAS: Php_TR69681.

10.7717/peerj.11135/supp-3 Supplemental Information 3 Melting curves of the reference genes. (A) Actina (ACT). (B) Elongation factor (EF-1α). (C) Tubulin (TUB). (D) Pyrophosphatase (PPA2).

10.7717/peerj.11135/supp-4 Supplemental Information 4 Phenotypic response to *Foph* from two cape gooseberry genotypes. Susceptible genotype: 09U128-5, Tolerant genotype: 09U140-5.

10.7717/peerj.11135/supp-5 Supplemental Information 5 Retrieved Illumina reads for transcriptomic assembly of *P. peruviana’s* stem and root tissues. Reads were downloaded from the Sequence Read Archive (SRA) under BioProject ID 67621.

10.7717/peerj.11135/supp-6 Supplemental Information 6 Disease severity scale used for the evaluation of vascular wilt in *Physalis peruviana* caused by *Fusarium oxysporum* f. sp. *physali*.

10.7717/peerj.11135/supp-7 Supplemental Information 7 Summary of reads and filtering process used for de novo transcriptome assembly.

10.7717/peerj.11135/supp-8 Supplemental Information 8 Summary of results from the cape gooseberry de novo assembly using the Trinity software.

10.7717/peerj.11135/supp-9 Supplemental Information 9 Differential expressed genes between *F. oxysporum* inoculated and mock-inoculated plants. Yellow color represents the 10 candidate DGEs genes selected.

10.7717/peerj.11135/supp-10 Supplemental Information 10 BLAST results from the primer pairs designed for the 10 DEGs and the de novo cape gooseberry transcriptome. Yellow color represents the original alignment with the *Physalis* transcript ID from the DEGs candidate genes (see Supplemental Table S5)

10.7717/peerj.11135/supp-11 Supplemental Information 11 Raw data from qRT-PCR experiment.

10.7717/peerj.11135/supp-12 Supplemental Information 12 DEGs and GWAS genes.

## Additional Information and Declarations

### Data Availability

The following information was supplied regarding data availability: Raw data are available in the Supplemental Files.
