# Supplementary material for: Combining transcriptome analysis and GWAS for identification and validation of marker genes in the Physalis peruviana-Fusarium oxysporum pathosystem
Source: PeerJ. 2021 Mar 22;9:e11135. doi: 10.7717/peerj.11135 (PMC7993016; doi:10.7717/peerj.11135)
Supplement: Supplemental Information 5 — Reads were downloaded from the Sequence Read Archive (SRA) under BioProject ID 67621. [file peerj-09-11135-s005.docx]

**Supplemental Table S1: Retrieved Illumina reads for transcriptomic assembly of *P. peruviana’s* stem and root tissues.** Reads were downloaded from the Sequence Read Archive (SRA) under BioProject ID 67621.

| **SRA accession number** | **Number of reads** |
| --- | --- |
| SRX971469 | 13,703,409 |
| SRX972116 | 2,569,399 |
| SRX978916 | 21,845,419 |
| SRX980678 | 3,570,574 |
| SRX981060 | 21,111,182 |
| SRX981416 | 17,763,564 |
| SRX983104 | 14,951,801 |
| SRX984241 | 2,856,971 |
