# Supplementary material for: Combining transcriptome analysis and GWAS for identification and validation of marker genes in the Physalis peruviana-Fusarium oxysporum pathosystem
Source: PeerJ. 2021 Mar 22;9:e11135. doi: 10.7717/peerj.11135 (PMC7993016; doi:10.7717/peerj.11135)
Supplement: Supplemental Information 6 [file peerj-09-11135-s006.docx]

**Supplemental Table S2:** **Disease severity scale used for the evaluation of vascular wilt in *Physalis peruviana* caused by *Fusarium oxysporum* f. sp. *physali.***

| **Degree** | **Disease Description** |
| --- | --- |
| 0 | Plant without symptoms. |
| 1 | Very limited wilting (no more than 10%) with slight chlorosis in leaves. |
| 2 | Limited wilting (10% to 30%) with few chlorotic leaves. |
| 3 | Severe wilting (30% to 90%) with some chlorotic leaves. |
| 4 | Very severe wilting (more than 90%) with defoliation. |
| 5 | Dead plant. |
