# Supplementary material for: Combining transcriptome analysis and GWAS for identification and validation of marker genes in the Physalis peruviana-Fusarium oxysporum pathosystem
Source: PeerJ. 2021 Mar 22;9:e11135. doi: 10.7717/peerj.11135 (PMC7993016; doi:10.7717/peerj.11135)
Supplement: Supplemental Information 7 [file peerj-09-11135-s007.docx]

**Supplemental Table S3: Summary of reads and filtering process used for *de novo* transcriptome assembly.**

|  | **Raw reads** | | **Reads after filtering process** | **Reads after normalization process** |
| --- | --- | --- | --- | --- |
| **Sequence count** | 98,372,319 | 87,488,110 | | 17,664,763 |
| **Total nucleotides (bp)** |  | | 8,089,957,428 | 1,621,835,415 |
| **Average length (bp)** | 100 | | 92.46 | 91 |

bp: base pairs
