# Supplementary material for: Combining transcriptome analysis and GWAS for identification and validation of marker genes in the Physalis peruviana-Fusarium oxysporum pathosystem
Source: PeerJ. 2021 Mar 22;9:e11135. doi: 10.7717/peerj.11135 (PMC7993016; doi:10.7717/peerj.11135)
Supplement: Supplemental Information 8 [file peerj-09-11135-s008.docx]

**Supplemental Table S4:** **Summary of results from the cape gooseberry *de novo* assembly using the Trinity software.**

|  | **All transcripts** | **Transcripts > 500 bp** | **Transcripts after *F. oxysporum* filtering**  **(> 500 bp)** |
| --- | --- | --- | --- |
| **Number** | 125,590 | 60,934 | 59,476 |
| **N50 size (bp)** | 1,207 | 1,511 | 1,523 |
| **Average length** | 779.6 | 1,263.30 | 1,271 |

bp: base pairs
